# Sex differences in characteristics and outcome in acute coronary syndrome patients in the Netherlands

**DOI:** 10.1007/s12471-019-1271-0

**Published:** 2019-04-15

**Authors:** M. E. ten Haaf, M. Bax, J. M. ten Berg, J. Brouwer, A. W. van’t Hof, R. J. van der Schaaf, P. R. Stella, R. M. Tjon Joe Gin, P. A. Tonino, A. G. de Vries, F. Zijlstra, E. Boersma, Y. Appelman

**Affiliations:** 1 Department of Cardiology, Amsterdam UMC, VU University Amsterdam, Amsterdam, The Netherlands; 2 The Netherlands Heart Institute, Utrecht, The Netherlands; 3 Department of Cardiology, HAGA Hospital, The Hague, The Netherlands; 4 Department of Cardiology, St. Antonius Hospital, Nieuwegein, The Netherlands; 5 Department of Cardiology, Medical Center Leeuwarden, Leeuwarden, The Netherlands; 6 Department of Cardiology, MUMC, Maastricht, The Netherlands; 7 Department of Cardiology, Zuyderland MC, Heerlen, The Netherlands; 8 Department of Cardiology, Onze Lieve Vrouwe Gasthuis location East, Amsterdam, The Netherlands; 9 Department of Cardiology, University Medical Center Utrecht, Utrecht, The Netherlands; 10 Department of Cardiology, Rijnstate Hospital, Arnhem, The Netherlands; 11 Department of Cardiology, Catharina Hospital, Eindhoven, The Netherlands; 12 Department of Cardiology, Albert Schweitzer Hospital, Dordrecht, The Netherlands; 13 Department of Cardiology, Erasmus MC, Rotterdam, The Netherlands

**Keywords:** Sex differences, Acute coronary syndrome, Registry

## Abstract

**Background:**

Sex differences in acute coronary syndrome (ACS) have been reported, but little is known about the situation in the Netherlands.

**Methods:**

This registry is a merge of available data on ACS patients in the electronic data capture systems of 11 centres with 24/7 interventional cardiology services. We included patients >18 years undergoing a cardiac catheterisation between 2010–2012. We evaluated sex differences in clinical and procedural characteristics and 1‑year mortality.

**Results:**

A total of 29,265 ACS patients (8,720 women and 20,545 men) were registered. Women were on average 4.5 years older (68.5 vs 63.0 years, *p* < 0.001) and had a higher prevalence of hypertension (62.7 vs 49.8%, *p* < 0.001) and insulin-dependent diabetes mellitus (9.6 vs 6.8%, *p* < 0.001) than men. Women less often presented with ST-elevation myocardial infarction (43.7% vs 47.6%, *p* < 0.001) and appeared to have less extensive coronary artery disease than men. Women less often underwent coronary angiography by radial access (52.5 vs 55.9%, *p* < 0.001). One-year mortality was higher in women than in men (7.3% and 5.6%, *p* < 0.001). More specific, the relationship between sex and mortality was age-dependent and showed higher mortality in women ≤71 years, but lower mortality in older women compared with men (*p*-interaction <0.001).

**Conclusion:**

We found differences in clinical and procedural characteristics and outcome between women and men admitted for ACS, which are in line with other Western countries. The limitations of our registry, based on existing local databases, can be overcome by the use of the prospective Netherlands Heart Registry that is currently in development.

**Electronic supplementary material:**

The online version of this article (10.1007/s12471-019-1271-0) contains supplementary material, which is available to authorized users.

## What’s new?

- To the best of our knowledge, this is the first time that data of a sizeable cohort of Dutch ACS patients are presented on sex differences.
- We merged and analysed data of almost 30,000 ACS patients who were treated in a representative selection of PCI-capable centres in the Netherlands.
- We found differences in clinical and procedural characteristics and outcome between women and men admitted for ACS, which are in line with other Western countries.
- The relation between sex and mortality appeared age-dependent, with higher mortality in women at a younger age, in particular in those presenting with STEMI, and lower mortality at advanced age—the turning point was 71 years.

## Introduction

In the Netherlands, cardiovascular disease (CVD) was responsible for 25.9% and 24.9% of deaths in women and men, respectively, in 2017 [1]. Myocardial infarction (MI), the most common entity of the acute coronary syndrome (ACS), represents 11.0% and 15.6% of cardiovascular deaths in women and men respectively [1]. An increasing amount of patients with ACS are women, due to the ageing of the population, changing risk profiles and shifts within diagnostic capabilities [2]. Several observational studies found that women presenting with ACS have a different clinical presentation than men and are managed differently [3, 4]. Conflicting results have been revealed about outcome differences between women and men [5]. Poorer in-hospital clinical outcome has been observed in women following ST-elevation myocardial infarction (STEMI), especially at younger age, although less severe disease has been observed with coronary angiography [6].

It is still unclear if these differences can solely be explained by sex or that they are related to differences in age, extent or impact of CVD risk factors, presentation or treatment. Also, whether the above-mentioned observations can be extrapolated to the situation in the Netherlands remains uncertain. We aimed to fill this knowledge gap, with particular focus on ACS patients. Although a national data registry for ACS patients is currently in preparation, for now a database containing representative, nationwide data is still lacking. So far, only one study reports sex differences on a multi-centre experience in the Netherlands, based on small patient numbers [7]. We realised, however, that a wide variety of information on sizeable sets of ACS patients is available in the electronic patient record systems of each (interventional) hospital in the Netherlands. We merged these data(sets), which created a large (virtual and retrospective) registry that was then available for analyses of sex differences.

## Methods

### Patients and material

This is an observational, retrospective study of ACS patients treated in the Netherlands. We included patients aged 18 years and older undergoing cardiac catheterisation for ACS in the period 2010–2012. We used data from existing electronic databases in each of the individual interventional cardiac centres. At the time we started our study, 30 interventional cardiac centres existed in the Netherlands. The 24 centres with electronically available clinical data and an established interventional cardiology programme that offered full 24/7 service were asked to participate. For several reasons, 13 eligible centres were unable or unwilling to participate, and thus we present data from a total of 11 centres.

Data were extracted in each individual centre using a pre-specified list of variables, which was mainly based on quality of care indicators [8, 9]. Electronically abstracted data included patient demographics, cardiovascular risk factors and history and characteristics of cardiac procedures. In patients who underwent multiple procedures within the study period, only the clinical information of the first procedure was retained. For the purpose of this study, patients were not contacted, subjected to acts, and neither was any mode of behaviour imposed, otherwise than as part of their regular treatment. Data extracted from the medical records were processed anonymously, not traceable to individual patients. Therefore, in accordance with Dutch law, written informed consent was not required.

### Definitions

To enable data merging of different centres, broad definitions were used. We distinguished STEMI, non-STEMI (NSTEMI) and unstable angina pectoris (UAP) as ACS subclasses, according to the European Society of Cardiology guidelines [10]. Prevalent risk factors including hypertension, hypercholesterolaemia, diabetes mellitus, including insulin dependence, smoking and family history of cardiovascular disease were defined as such in the dataset of the concerning centre. This also applies to prior MI, percutaneous coronary intervention (PCI), coronary artery bypass grafting (CABG) and renal failure. Left ventricular ejection fraction (LVEF) was defined as good (>50%), moderately impaired (30–50%) and severely impaired (<30%). Segment involvement was created using the coronary artery segments mapping on the coronary angiogram according to the American Heart Association classification [11]. Severity of coronary artery disease (CAD) was expressed in number of segments and vessels involved with ≥50% stenosis. Multi-vessel obstructive disease was defined as ≥50% stenosis in left main artery or ≥50% stenosis in ≥2 separate epicardial coronary artery territories.

#### Clinical study endpoint

The clinical study endpoint was all-cause mortality, which we report at 7‑day, 30-day and 1‑year follow-up. Survival status for each patient was obtained from municipal civil registries.

### Statistical analysis

The availability of specific data items varied between the participating centres. Descriptive statistics were provided for all available data. Inferential statistics (including statistical modelling) was limited to variables that were available for at least 4 participating centres.

Continuous variables were expressed as means with standard deviations and medians with interquartile ranges. Categorical variables are presented as numbers with percentages. We used Mann-Whitney tests (continuous variable) and chi-squared tests (categorical) to evaluate differences between women and men.

Estimates of the cumulative incidence of mortality were obtained by the method of Kaplan-Meier, and differences between women and men were evaluated by the log-rank test. The relationship between sex and 1‑year mortality was further analysed using Cox proportional hazards regression models. We applied univariable and multivariable analyses, with adjustment for age and further adjustment for 1, potential, confounder, including body mass index (BMI), hypertension, hypercholesterolaemia, diabetes mellitus, smoking, family history of CVD, prior MI, prior PCI, prior CABG, LVEF, renal failure, acute MI, STEMI, access site, multi-vessel disease, segment involvement, multi-lesion procedure, multi-vessel procedure and use of drug-eluting stent. Full adjustment for multiple (potential) confounders was not possible as structural missing data precluded multiple imputation. We introduced a sex*age interaction term in the Cox model to study the relationship of sex, age and 1‑year mortality. As results showed a relevant sex*age interaction the clinical endpoints were also presented in 5 strata according to age. Sex differences were analysed in the entire ACS study population and after categorisation for STEMI and NSTEMI/UAP patients.

SPSS software, version 24, was used to perform all analyses. Confidence intervals were calculated at the 95% level. A *p*-value below 0.05 (two-sided test) was considered statistically significant.

## Results

### Patients

**Fig. 1:**
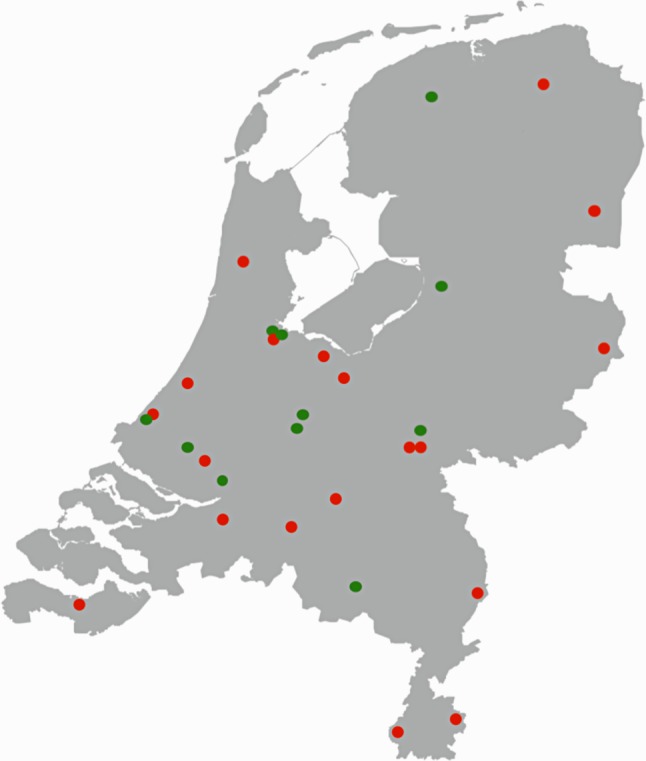
Geographical distribution of participating interventional centres. Geographical distribution of participating interventional centres. *Green dots* represent participating centres, *red dots* represent non-participating centres

A total of 8,720 (29.8%) women and 20,545 (70.2%) men with ACS followed by coronary angiography were included. The number of patients included per centre ranged from 1,184 to 5,592. The participating centres had a widespread geographical distribution (Fig. 1).

### Baseline characteristics

**Fig. 2:**
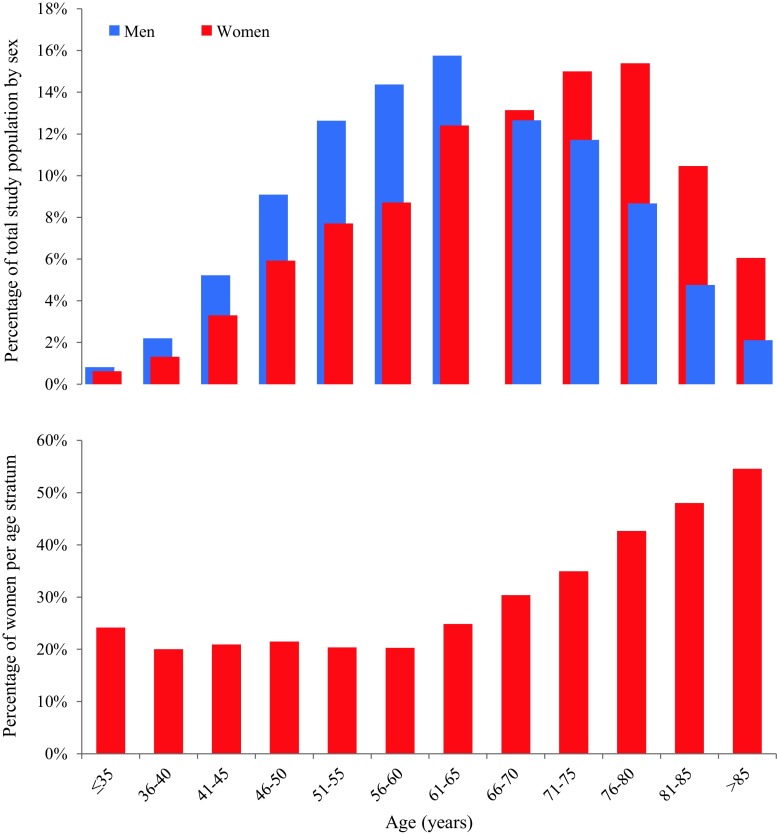
Study patients according to sex and age. Study patients by sex and age. *Upper part* shows percentage of the total study sample of ACS patients by sex, *bottom part* shows percentage of women per age stratum. *ACS* acute coronary syndrome

Women and men had a different age distribution (Fig. 2). Women were on average 4.5 years older (mean age 68.5 vs 63.0 years, *p* < 0.001) and had a higher prevalence of traditional risk factors for CVD, including hypertension (62.7 vs 49.8%, *p* < 0.001) and insulin-dependent diabetes mellitus (9.6 vs 6.8%, *p* < 0.001), but a lower prevalence of current smoking (27.2 vs 36.0%, *p* < 0.001), family history of CAD (40.2 vs 42.1%, *p* = 0.037) and history of MI, PCI or CABG. Almost half of the patients had hypercholesterolaemia and about 5% of the patients had a severely impaired LVEF (Tab. 1). Renal failure was comparable between both groups with 7.4 vs 7.1% (*p* = 0.61).

**Table 1:** Baseline characteristics of ACS patients included in the registry; Merged data according to sex

	**Women**	**Men**	***P-value***
	*N* = 8,720 (29.8)	*N* = 20,545 (70.2)	
*Demographics*			
Age^a^	68.5 (12.7)	63.0 (12.0)	<0.001
BMI^b^	27.0 (5.0)	27.2 (3.9)	0.15
*Presentation*			
Systolic blood pressure^c^	132 (30)	126 (26)	<0.001
Diastolic blood pressure^d^	74 (17)	75 (16)	0.063
*Risk factors*			
Hypertension	2,909/4,637 (62.7)	5,535/11,107 (49.8)	<0.001
Hypercholesterolaemia	1,896/4,128 (45.9)	4,718/10,021 (47.1)	0.21
Diabetes mellitus	1,300/6,363 (20.4)	2,516/15,285 (16.5)	<0.001
Diabetes mellitus type			
– Insulin-dependent	213/2,218 (9.6)	364/5,335 (6.8)	<0.001
Current smoker	981/3,604 (27.2)	3,099/8,598 (36.0)	<0.001
Family history of cardiac disease	1,760/4,376 (40.2)	4,501/10,700 (42.1)	0.037
*Cardiovascular history*			
Prior MI	965/5,113 (18.9)	2,924/12,416 (23.6)	<0.001
Prior PCI	850/4,603 (18.5)	2,302/10,885 (21.1)	<0.001
Prior CABG	376/6,123 (6.1)	1,283/14,790 (8.7)	<0.001
*LVEF*			0.22
>50%	1,270/1,525 (83.3)	2,906/3,513 (82.7)	
30–50%	187/1,525 (12.3)	410/3,513 (11.7)	
<30%	68/1,525 (4.5)	197/3,513 (5.6)	
*Non-cardiovascular history*			
Renal failure	247/3,350 (7.4)	594/8,366 (7.1)	0.61

*ACS* acute coronary syndrome, *BMI* body mass index, *MI* myocardial infarction, *PCI* percutaneous coronary intervention, *CABG* coronary artery bypass grafting, *LVEF* left ventricular ejection fraction. Continuous variables are expressed as mean (standard deviation) values, categorical variables as counts of the total data available in women and men (percentage). Percentages may not add to 100 due to rounding

^a^Data were available in 7,701 women, 18,372 men

^b^Data were available in 3,612 women, 8,919 men

^c^Data were available in 1,914 women, 4,758 men

^d^Data were available in 1,914 women, 4,758 men

### Procedural characteristics

Procedural data are listed in Tab. 2. A total of 65.1% women and 68.2% men with ACS underwent coronary angiography for acute MI, of which significantly less women (43.7%) than men (47.6%) were treated for STEMI. Fewer women than men underwent coronary angiography by radial access (52.5 vs 55.9%). Women had significantly less extensive CAD with relatively more single-vessel disease compared with men. Most lesions were located in the left anterior descending coronary artery (49.9%), followed by the right coronary artery (41.1%) in both sexes. In women, relatively fewer stents (per treated segment and in total) were implanted than in men (46.2 vs 49.4%). Drug-eluting stents were the most commonly implanted stents in both women and men.

**Table 2:** Procedural characteristics of ACS patients included in the registry; Merged data according to sex

	Women	Men	*P-value*
	*N* = 8,720 (29.8)	*N* = 20,545 (70.2)	
*Indication*			
AMI	4,478/6,882 (65.1)	11,063/16,217 (68.2)	<0.001
*ACS subtype*			<0.001
STEMI	2,462/5,635 (43.7)	6,123/12,852 (47.6)	
NSTEMI	933/5,635 (16.6)	1,995/12,852 (15.5)	
Unstable angina	2,240/5,635 (39.8)	4,734/12,852 (36.8)	
*Access site*			<0.001
Radial	2,831/5,393 (52.5)	7,180/12,843 (55.9)	
Femoral	2,287/5,393 (42.4)	5,041/12,843 (39.3)	
Other	275/5,393 (5.1)	622/12,843 (4.8)	
*Severity CAD*			<0.001
No CAD	190/5,583 (3.4)	313/13,653 (2.3)	
Single-vessel	2,756/5,583 (49.4)	6,406/13,653 (46.9)	
Multi-vessel^a^	2,637/5,583 (47.2)	6,934/13,653 (50.8)	
*Segment involvement*			
RCA	2,292/5,372 (42.7)	5,419/13,411 (40.4)	0.004
LAD	2,755/5,378 (51.2)	6,629/13,424 (49.4)	0.022
CX	1,384/5,372 (25.8)	4,078/13,426 (30.4)	<0.001
Left main	174/5,348 (3.3)	450/13,356 (3.4)	0.69
Graft	35/3,464 (1.0)	191/8,069 (2.4)	<0.001
*Number of segments* ^b^	1 (1–2)	1 (1–2)	0.54
*Multi-segment procedure*	1,323/4,730 (28.0)	3,251/11,393 (28.6)	0.45
*Multi-vessel procedure*	1,081/5,355 (20.2)	2,799/13,376 (20.9)	0.26
*Implanted stent type*			0.15
DES	2,297/4,031 (57.0)	5,765/10,144 (56.8)	
BMS	1,042/4,031 (25.8)	2,602/10,144 (25.7)	
Combination of DES and BMS	425/4,031 (10.5)	1,002/10,144 (9.9)	
Other (including BVS)	267/4,031 (6.6)	775/10,144 (7.6)	

*ACS* acute coronary syndrome, *AMI* acute myocardial infarction, *(N)STEMI* (non‑)ST-elevation myocardial infarction, *CAD* coronary artery disease, *RCA* right coronary artery, *LAD* left anterior descending artery, *CX* circumflex, *DES* drug-eluting stent, *BMS* bare-metal stent, *BVS* bioresorbable vascular scaffold. Continuous variables are expressed as median (interquartile range) values, categorical variables as counts of the total data available in women and men (percentage). Percentages may not add to 100 due to rounding

^a^Including two-vessel, three-vessel and left main disease

^b^Data was available in 4,401 women, 10,804 men

### Mortality

**Fig. 3:**
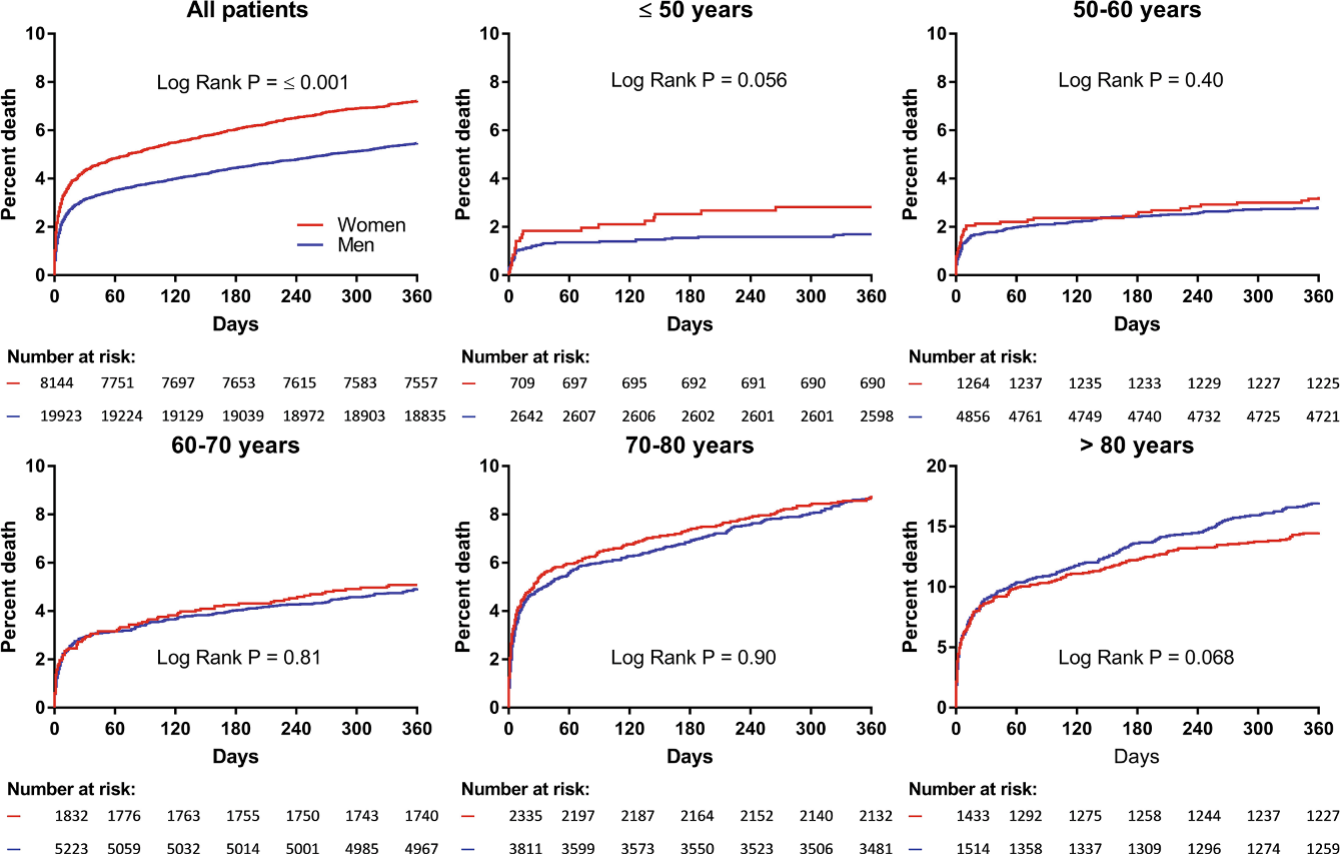
Kaplan-Meier curves for all-cause mortality according to sex and age stratum up to 1‑year follow-up. Kaplan-Meier curves for all-cause mortality in ACS patients according to sex and age stratum up to 1‑year follow-up. *ACS* acute coronary syndrome

**Fig. 4:**
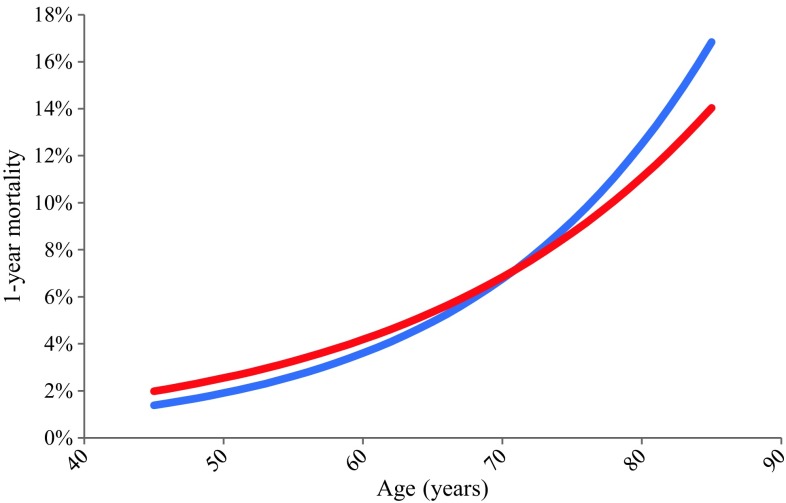
Adjusted all-cause 1‑year mortality of the sex*age interaction. Adjusted all cause 1‑year mortality of the sex and age interaction. *Red line* represents women, *blue line* represents men with ACS. *ACS* acute coronary syndrome

Women had a higher cumulative incidence of all-cause mortality than men (7.3% vs 5.6%, hazard ratio [HR] 1.31 and 95% confidence interval [CI] 1.18–1.45, *p* < 0.001). After adjustment for age, the observed mortality difference disappeared. However, the sex*age*mortality relation appeared more complex, as we found a statistically significant interaction between age and sex with respect to 1‑year mortality (*p* < 0.001). At younger age, women had a higher mortality than men, whereas this pattern was reversed at elderly age (Fig. 3). Based on the logistic regression model, the age of 71 years appeared to be the turning point (Fig. 4). After adjustment for age, the sex*age interaction term and single variables, female sex was still associated with higher mortality (data not shown).

### STEMI versus NSTEMI/UAP

Sex-related differences in baseline and the majority of procedural characteristics of STEMI and NSTEMI/UAP patients were comparable with the entire ACS population (Supplementary data—Tables 1–3). Compared with STEMI patients, the risk profile of NSTEMI patients was more unfavourable, except for the risk factor smoking. Women and men with NSTEMI were respectively 1.2 and 3.0 years older, had a 21.2 and 22.8% higher incidence of hypertension, 24.5 and 26.7% higher incidence of hypercholesterolaemia, 10.1 and 8.5% higher incidence of diabetes mellitus and 6.0 and 6.7% more renal failure. The prevalence of current smoking was 16.3% lower for women and 14.5% lower for men with NSTEMI. Both women and men with NSTEMI more frequently had a prior cardiovascular history and had on average a worse LVEF.

In women and men with STEMI, a femoral approach was relatively more often applied than in NSTEMI patients. There were no significant sex-related differences in CAD severity in STEMI patients. However, in NSTEMI patients, women more often had non-obstructive and single-vessel disease.

In all age categories, STEMI patients had higher 1‑year mortality than NSTEMI patients. Women with STEMI had higher 1‑year mortality than men (9.4% and 6.8%; respectively *p* < 0.001). The difference was most striking at young (≤50 years) age (4.8% and 2.3%, respectively; *p* = 0.022) and was seen from 30-day follow-up. Women and men with NSTEMI had similar mortality (5.0% and 4.3%, respectively; *p* = 0.15).

## Discussion

We merged and analysed data of almost 30,000 ACS patients who had been treated in a representative selection of PCI-capable centres in the Netherlands. To the best of our knowledge this is the first time that data of a sizeable cohort of Dutch ACS patients are presented. Similar to other countries [12], we found relevant sex differences in clinical and procedural characteristics and outcome. Women were older and more often had prevalent cardiovascular risk factors, including hypertension and diabetes mellitus. Women nevertheless had less extensive macrovascular CAD. The relation between sex and mortality appeared age-dependent, with higher mortality in women at younger age, in particular in those presenting with STEMI, and lower mortality at advanced age—the turning point was 71 years.

The women:man ratio in our study was 1:2.4, which is largely in agreement with the Swedish Coronary Angiography and Angioplasty Registry (SCAAR) registry data on ACS patients (ratio 1:2.7) and the French Registry of Acute ST-elevation or non-ST-elevation Myocardial Infarction (ratio 1:2.8) [12, 13]. In view of these international studies, and since we included ‘all-comers’ in participating hospitals that can be considered representative for all interventional centres in the Netherlands, we conclude that fewer women than men present with ACS necessitating angiography. Still, we are aware that we only enrolled patients who reached the cath lab, which might have resulted in a somewhat underestimated incidence of ACS in women. Several studies have shown that women are insufficiently aware of indicative symptoms, and seek medical care later than men [14, 15], which might be fatal in the case of ACS. In addition, studies suggested that (especially younger) women might present with complaints that are not suggestive for macrovascular obstruction [16]. Consequently, these women do not reach the cath lab. Unfortunately, we cannot quantify the magnitude of these effects on the basis of our study data.

We included consecutive ACS patients, and found that women were on average 4 years older than men. They had less extensive CAD, despite having more comorbid risk factors including hypertension and diabetes mellitus, which is largely in accordance with previous studies [17, 18]. It is known that women experience the onset of obstructive CAD approximately 6–8 years later than men, and that they are older when admitted for their first MI [19]. The most widely accepted explanation for this phenomenon is that pre-menopausal women are largely protected against obstructive CAD by circulating oestrogen [20]. The cardiovascular risk profile of women worsens once menopause is over, and the prevalence of chronic CAD then steeply increases with age, as does the incidence of acute expressions of the disease.

Women (especially those with NSTEMI) had a higher incidence of non-obstructive CAD [NOCAD] than men. It was previously reported that MI with non-obstructive coronary arteries (MINOCA) is more often seen in women and in patients with NSTEMI. Recent publications argue that MINOCA should be interpreted as a working diagnosis in order to find the true underlying pathophysiologic mechanism [21]. In women several mechanisms seem to be responsible for MINOCA, such as differences in plaque composition, with women having less plaque burden but more plaque erosion and thin-cap fibroatheromas [17]. Other mechanisms include coronary artery dissection, coronary vasospasm (including microvascular), Takotsubo cardiomyopathy and myocarditis. Dedicated studies with invasive diagnostic measurements are necessary to disentangle this complex phenomenon [21, 22].

Our mortality data were similar to the large combined UK/Swedish ACS registry by Kunadian et al. [19]. In view of the worse risk profile and extent of coronary obstructions, one would expect more favourable mortality figures in women than in men, but results showed the opposite. Several explanations are postulated for the higher mortality rates in women with ACS, including lower revascularisation rates and lower guideline adherence. Recently, Alabas et al. found higher mortality rates in women with STEMI compared with men, but not in women with NSTEMI compared with men after correction for treatment according to the guidelines [23]. Possibly delayed presentation, lower rate of referral for angiography or bleeding complications play a role in the higher mortality rates in women [5]. Another explanation might be a higher incidence of MINOCA, which may limit clinical attention and is associated with adverse clinical outcome [22]. Unfortunately, our registration does not contain sufficient information to disentangle these phenomena in detail.

Interestingly, the excess mortality in women was particularly striking in younger STEMI patients—in fact, elderly women had a lower mortality rate than men. This finding was also observed by various previous studies [24, 25]. Cardiovascular risk factors, including overweight and smoking, are increasingly prevalent among young women and seem to have a differential impact on endothelial and microvascular dysfunction than in men, and thus on the development of CAD [26]. Young women tend to have more plaque erosion while older women have plaque rupture [27]. Also, young women have a higher frequency of alternative aetiologies, including spontaneous coronary artery dissection and coronary vasospasm. Finally, younger women are less likely to have chest pain and discomfort, and more likely to have atypical presentations, potentially leading to under-recognition or delayed presentation of STEMI, making acute reperfusion less likely [28]. The excess mortality in older men can possibly be explained by the longer exposure to obstructive coronary disease and shorter life expectancy within men.

### Data collection

We were able to collect and merge data of ACS patients of 11 experienced interventional centres with a widespread geographical distribution, and thus to construct a large data set that is unique for the Netherlands. The majority of the remaining 13 centres were sympathetic, but were not able to provide data. They had switched to a new electronic patient record system, collected data in free text fields or had no ability to extract data from the data system. Unlike in Sweden, until now Dutch centres have freedom of choosing their own electronic patient record system, which hampers data sharing. Some of the centres understandably refused participation because data were not validated or registered for scientific purposes. It took a disproportionate amount of time and energy to validate and merge datasets, whereas missing data was a serious concern. We recognise that for more detailed analyses—for example analyses of time trends—uniform, prospective patient cohorts are necessary. Clinical registries can be very useful for outcome monitoring and benchmarking with other European countries in order to improve the quality of care. In addition, clinical registries have proven their value in multi-centre observational research, and in registry-based randomised clinical trials [29, 30]. The Netherlands Society of Cardiology recognised that we should no longer lag behind other European countries, and initiated a national ACS registry. We expect the first results within 5 years from now.

### Limitations

Several limitations of our work warrant discussion. The retrospective design represents a serious drawback, but also highlights a sobering fact: a proper, prospective, nationwide ACS registry is still lacking in the Netherlands. We validated data (definitions and outliers) at hospital level, but validation on patient level was not feasible. We are aware of the selection bias that is created by studying only patients admitted to the cath lab, and thus neglecting conservatively treated patients. Remarkably and inexplicably, we found more missing data in women than in men. Due to missing data, we were not able to perform a full multivariable regression analysis. Also, we could not report on details of clinical and procedural characteristics, including angiographic data and time intervals. Especially time intervals would have been very interesting in view of previous described sex differences in the literature and the excellent logistics concerning STEMI care in the Netherlands. All in all, given that 46% of interventional hospitals participated, with satisfactory geographical distribution, and given the large sample size, our data provide a reasonable insight in ACS management, and sex differences in ACS management, in our country. It is reassuring to learn that our findings were similar to other national registries, including the United Kingdom and Sweden.

## Conclusion

In this retrospective study, based on data from a representative selection of PCI-capable centres in the Netherlands, differences were observed in clinical and procedural characteristics and in outcome between women and men presenting with ACS and admitted for coronary angiography, which are similar to other Western countries. Female sex was associated with higher all-cause mortality up to one year after ACS admission in young patients, especially in those presenting with STEMI. Data provided from registries are important to improve the quality of care of the entire population of ACS patients, but especially in groups that are underrepresented in clinical trials, including women. Our study underscores the importance of a national registry of ACS patients in order to monitor treatment quality and trends in treatment quality, patient outcome, as well as for benchmarking purposes.

## Caption Electronic Supplementary Material

Table 1: Baseline characteristics; Merged data according to sex and indication procedure

Table 2: Procedural characteristics; Merged data according to sex and indication procedure

Table 3: All-cause mortality* up to 1 year; Merged data according to sex, age stratum and indication procedure
