# Supplementary material for: Sex differences in characteristics and outcome in acute coronary syndrome patients in the Netherlands
Source: Neth Heart J. 2019 Apr 15;27(5):263–71. doi: 10.1007/s12471-019-1271-0 (PMC6470244; doi:10.1007/s12471-019-1271-0)
Supplement: Supplementary file 1 — Table 1: Baseline characteristics; Merged data according to sex and indication procedure [file 12471_2019_1271_MOESM1_ESM.docx]

**Supplementary data – Table 1 - Baseline characteristics; Merged data according to sex and indication procedure**

|  |  | **STEMI** | |  | **NSTEMI/UAP** | |  |
| --- | --- | --- | --- | --- | --- | --- | --- |
|  |  | **Women** | **Men** | ***P-value*** | **Women** | **Men** | ***P-value*** |
|  |  | *N*=2462 (28.7) | *N*=6123 (71.3) |  | *N*=3173 (32.0) | *N*=6729 (68.0) |  |
| *Demographics* |  |  |  |  |  |  |  |
| Age* |  | 68.0 (13.5) | 61.8 (12.2) | <0.001 | 69.2 (12.0) | 64.8 (11.7) | <0.001 |
| BMI† |  | 26.7 (5.2) | 26.9 (3.8) | 0.29 | 27.4 (5.0) | 27.4 (4.1) | 0.99 |
| *Presentation* |  |  |  |  |  |  |  |
| SBP‡ |  | 131 (30) | 127 (26) | 0.003 | 139 (29) | 133 (25) | <0.001 |
| DBP∫ |  | 75 (17) | 76 (17) | 0.15 | 76 (17) | 78 (17) | 0.040 |
| *Risk factors* |  |  |  |  |  |  |  |
| Hypertension |  | 533/1106 (48.2) | 925/2650 (34.9) | <0.001 | 1243/1792 (69.4) | 2157/3780 (57.1) | <0.001 |
| Hypercholesterolaemia |  | 212/913 (23.2) | 504/2222 (22.7) | 0.74 | 802/1679 (47.8) | 1767/3578 (49.4) | 0.27 |
| Diabetes mellitus |  | 236/1531 (15.4) | 433/3776 (11.5) | <0.001 | 604/2364 (25.5) | 1034/4932 (21.0) | <0.001 |
| Diabetes mellitus type |  |  |  |  |  |  |  |
| Insulin-dependent |  | 34/425 (8.0) | 62863 (7.2) | 0.60 | 136/1071 (12.7) | 225/2362 (9.5) | 0.005 |
| Current smoker |  | 376/1040 (36.2) | 1119/2520 (44.4) | <0.001 | 344/1729 (19.9) | 1037/3718 (27.9) | <0.001 |
| Family history of cardiac disease |  | 348/1048 (33.2) | 943/2589 (36.4) | 0.066 | 675/1717 (39.3) | 1536/3688 (41.6) | 0.10 |
| *Cardiovascular history* |  |  |  |  |  |  |  |
| Prior MI |  | 121/1266 (9.6) | 427/3224 (13.2) | 0.001 | 546/2083 (26.2) | 1473/4391 (33.5) | <0.001 |
| Prior PCI |  | 109/1087 (10.0) | 394/2761 (14.3) | <0.001 | 597/2228 (26.8) | 1463/4674 (31.3) | <0.001 |
| Prior CABG |  | 36/1343 (2.7) | 110/3396 (3.2) | 0.32 | 223/2367 (9.4) | 748/4941 (15.1) | <0.001 |
| LVEF |  |  |  | 0.64 |  |  | 0.070 |
| >50 % |  | 505/563 (89.7) | 1172/1303 (89.9) |  | 608/740 (82.2) | 1324/1643 (80.6) |  |
| 30-50 % |  | 29/563 (5.2) | 75/1303 (5.8) |  | 109/740 (14.7) | 233/1643 (14.2) |  |
| <30 % |  | 29/563 (5.2) | 56/1303 (4.3) |  | 23/740 (3.1) | 86/1643 (5.2) |  |
| *Non-cardiovascular history* |  |  |  |  |  |  |  |
| Renal failure |  | 18/316 (5.7) | 35/787 (4.4) | 0.38 | 152/1302 (11.7) | 312/2791 (11.2) | 0.64 |

*(N)STEMI* (non-)ST-segment elevation myocardial infarction, *UAP* unstable angina pectoris, *BMI* body mass index, *SBP* systolic blood pressure, *DBP* diastolic blood pressure, *MI* myocardial infarction, *PCI* percutaneous coronary intervention, *CABG* coronary artery bypass grafting, *LVEF* left ventricular ejection function. Continuous variables are expressed as mean (standard deviation) values, categorical variables as counts of the total data available in women and men (percentage).

STEMI: *Data were available in 2449 women, 6084 men; † Data were available in 875 women, 2143 men; ‡ Data were available in 743 women, 1788 men; ∫ Data were available in 743 women, 1788 men.
NSTEMI: *Data were available in 3150 women, 6689 men; † Data were available in 1323 women, 2973 men; ‡ Data were available in 559 women, 1220 men; ∫ Data were available in 559 women, 1220 men.
