# Supplementary material for: Sex differences in characteristics and outcome in acute coronary syndrome patients in the Netherlands
Source: Neth Heart J. 2019 Apr 15;27(5):263–71. doi: 10.1007/s12471-019-1271-0 (PMC6470244; doi:10.1007/s12471-019-1271-0)
Supplement: Supplementary file 2 — Table 2: Procedural characteristics; Merged data according to sex and indication procedure [file 12471_2019_1271_MOESM2_ESM.docx]

**Supplementary data – Table 2 - Procedural characteristics of ACS patients included in the registry; Merged data according to sex and indication procedure**

|  |  | **STEMI** | |  | **NSTEMI/UAP** | |  |
| --- | --- | --- | --- | --- | --- | --- | --- |
|  |  | **Women** | **Men** | ***P-value*** | **Women** | **Men** | ***P-value*** |
|  |  | *N*=2462 (28.7) | *N*=6123 (71.3) |  | *N*=3173 (32.0) | *N*=6729 (68.0) |  |
| *Access site* |  |  |  | 0.056 |  |  | 0.030 |
| Radial |  | 680/1669 (40.7) | 1874/4242 (44.2) |  | 953/966 (48.5) | 2106/4048 (52.0) |  |
| Femoral |  | 829/1669 (49.7) | 1982/4242 (46.7) |  | 899/1966 (45.7) | 1709/4048 (42.2) |  |
| Other |  | 160/1669 (9.6) | 386/4242 (9.1) |  | 114/1966 (5.8) | 233/4048 (5.8) |  |
| *Severity CAD* |  |  |  | 0.65 |  |  | <0.001 |
| No CAD |  | 80/1464 (5.5) | 190/3609 (5.3) |  | 89/1843 (4.8) | 83/3940 (2.1) |  |
| Single-vessel |  | 695/1464 (47.5) | 1669/3609 (46.2) |  | 888/1843 (48.2) | 1708/3940 (43.4) |  |
| Multi-vessel* |  | 689/1464 (47.1) | 1750/3609 (48.5) |  | 866/1843 (47.0) | 2149/3940 (54.5) |  |
| *Segment involvement* |  |  |  |  |  |  |  |
| RCA |  | 639/1421 (45.0) | 1468/3569 (41.1) | 0.013 | 578/1657 (34.9) | 1210/3728 (32.5) | 0.081 |
| LAD |  | 591/1413 (41.8) | 1482/3577 (41.4) | 0.80 | 906/1669 (54.3) | 1801/3729 (48.3) | <0.001 |
| CX |  | 209/1409 (14.8) | 689/3567 (19.3) | <0.001 | 489/1660 (29.5) | 1322/3736 (35.4) | <0.001 |
| Left main |  | 20/1404 (1.4) | 52/3545 (1.5) | 0.91 | 79/1652 (4.8) | 179/3704 (4.8) | 0.94 |
| Graft |  | 10/1616 (0.6) | 38/3937 (1.0) | 0.21 | 19/1452 (1.3) | 127/3107 (4.1) | <0.001 |
| Number of segments † |  | 1 (1-1) | 1 (1-1) | 0.038 | 1 (1-2) | 1 (1-2) | 0.26 |
| Multi-segment procedure |  | 263/1621 (16.2) | 743/3982 (18.7) | 0.031 | 691/1897 (36.4) | 1568/4153 (37.8) | 0.32 |
| Multi-vessel procedure |  | 58/1404 (4.1) | 186/3550 (5.2) | 0.10 | 394/1657 (23.8) | 855/3715 (23.0) | 0.54 |
| Implanted stent type |  |  |  | 0.089 |  |  | 0.35 |
| DES |  | 674/1146 (58.8) | 1672/2885 (58.0) |  | 739/1240 (59.6) | 1746/2861 (61.0) |  |
| BMS |  | 376/1146 (32.8) | 898/2885 (31.1) |  | 240/1240 (19.4) | 536/2861 (18.7) |  |
| Combination of DES and BMS |  | 12/1146 (1.0) | 49/2885 (1.7) |  | 156/1240 (12.6) | 312/2861 (10.9) |  |
| Other (including BVS) |  | 84/1146 (7.3) | 266/2885 (9.2) |  | 105/1240 (8.5) | 267/2861 (9.3) |  |

*ACS* acute coronary syndrome, *(N)STEMI* (non-)ST-segment elevation myocardial infarction, *CAD* coronary artery disease, *RCA* right coronary artery, *LAD* left anterior descending artery, *CX* circumflex, *DES* drug-eluting stent, *BMS* bare-metal stent. Continuous variables are expressed as mean (standard deviation) and median (interquartile range) values, categorical variables as counts of the total data available in women and men (percentage). Percentages may not add to 100 due to rounding.

* Including two-vessel, three-vessel and left main disease. † Data was available in 1480 women, 3695 men (STEMI) and in 1710 women, 3846 men (NSTEMI).
