# Supplementary material for: Sex differences in characteristics and outcome in acute coronary syndrome patients in the Netherlands
Source: Neth Heart J. 2019 Apr 15;27(5):263–71. doi: 10.1007/s12471-019-1271-0 (PMC6470244; doi:10.1007/s12471-019-1271-0)
Supplement: Supplementary file 3 — Table 3: All-cause mortality* up to 1 year; Merged data according to sex, age stratum and indication procedure [file 12471_2019_1271_MOESM3_ESM.docx]

**Supplementary data – Table 3 – All-cause mortality* up to 1 year of ACS patients included in the registry; Merged data according to sex and age stratum**

|  |  | **STEMI** | |  | **NSTEMI/UAP** | |  |
| --- | --- | --- | --- | --- | --- | --- | --- |
|  |  | **Women** | **Men** | ***P-value***† | **Women** | **Men** | ***P-value***† |
| All patients |  | *N*=2458 (28.7) | *N*=6104 (71.3) |  | *N*=3092 (32.1) | *N*=6538 (67.9) |  |
| All-cause mortality – 1y |  | 230 (9.4%) | 414 (6.8%) | <0.001 | 134 (5.0%) | 283 (4.3%) | 0.15 |
| All-cause mortality – 30d |  | 171 (7.0%) | 283 (4.6%) | <0.001 | 39 (1.9%) | 94 (1.4%) | 0.086 |
| All-cause mortality – 7d |  | 137 (5.6%) | 212 (3.5%) | <0.001 | 27 (0.9%) | 59 (0.9%) | 0.89 |
| **Patients ≤50 years** |  | 271 (20.3) | 1065 (79.7) |  | 223 (24.0) | 706 (76.0) |  |
| All-cause mortality – 1y |  | 13 (4.8%) | 24 (2.3%) | 0.022 | 2 (0.9%) | 3 (0.4%) | 0.40 |
| All-cause mortality – 30d |  | 11 (4.1%) | 19 (1.8%) | 0.024 | 0 | 2 (0.3%) | 0.43 |
| All-cause mortality – 7d |  | 8 (3%)) | 15 (1.4%) | 0.081 | 0 | 2 (0.3%) | 0.43 |
| **Patients >50-60 years** |  | 449 (20.3) | 1767 (79.7) |  | 478 (23.4) | 1563 (76.7) |  |
| All-cause mortality – 1y |  | 1.6 (3.6%) | 61 (3.5%) | 0.90 | 5 (1.0%) | 26 (1.7%) | 0.33 |
| All-cause mortality – 30d |  | 14 (3.1%) | 43 (2.4%) | 0.41 | 2 (0.4%) | 6 (0.4%) | 0.92 |
| All-cause mortality – 7d |  | 11 (2.4%) | 32 (1.8%) | 0.38 | 2 (0.4%) | 4 (0.3%) | 0.57 |
| **Patients >60-70 years** |  | 515 (24.1) | 1621 (75.9) |  | 796 (28.4) | 2004 (71.6) |  |
| All-cause mortality – 1y |  | 39 (7.6%) | 95 (5.9%) | 0.16 | 27 (3.4%) | 70 (3.5%) | 0.90 |
| All-cause mortality – 30d |  | 28 (5.4%) | 70 (4.3%) | 0.28 | 8 (1.0%) | 17 (0.8%) | 0.69 |
| All-cause mortality – 7d |  | 23 (4.5%) | 52 (3.2%) | 0.17 | 4 (0.5%) | 8 (0.4%) | 0.71 |
| **Patients >70-80 years** |  | 732 (38.6) | 1165 (61.4) |  | 986 (38.5) | 1575 (61.5) |  |
| All-cause mortality – 1y |  | 70 (9.6%) | 127 (10.9%) | 0.39 | 65 (6.6%) | 105 (6.7%) | 0.92 |
| All-cause mortality – 30d |  | 54 (7.4%) | 80 (6.9%) | 0.66 | 25 (2.5%) | 48 (3.0%) | 0.44 |
| All-cause mortality – 7d |  | 42 (5.7%) | 61 (5.2%) | 0.63 | 13 (1.3%) | 32 (2.0%) | 0.18 |
| **Patients >80 years** |  | 478 (51.7) | 447 (48.3) |  | 586 (47.4) | 650 (52.6) |  |
| All-cause mortality – 1y |  | 91 (19.0%) | 106 (23.7%) | 0.098 | 55 (9.4%) | 78 (12.0%) | 0.15 |
| All-cause mortality – 30d |  | 64 (13.4%) | 70 (15.7%) | 0.35 | 24 (4.1%) | 20 (3.1%) | 0.34 |
| All-cause mortality – 7d |  | 53 (11.1%) | 51 (11.4%) | 0.89 | 8 (1.4%) | 13 (2.0%) | 0.39 |

*ACS* acute coronary syndrome, *(N)STEMI*, (non-)ST-segment elevation myocardial infarction, *UAP* unstable angina pectoris. Continuous variables are expressed as number (percentage).
* Kaplan-Meier estimates. † Log-rank *P*-value.
